# Supplementary material for: Molecular detection and risk factors of Eimeria in native and exotic chickens under varying management systems in Bangladesh
Source: PLoS One. 2025 Jul 15;20(7):e0327037. doi: 10.1371/journal.pone.0327037 (PMC12262850; doi:10.1371/journal.pone.0327037)
Supplement: S1_raw_images — (PDF) [file pone.0327037.s001.pdf]

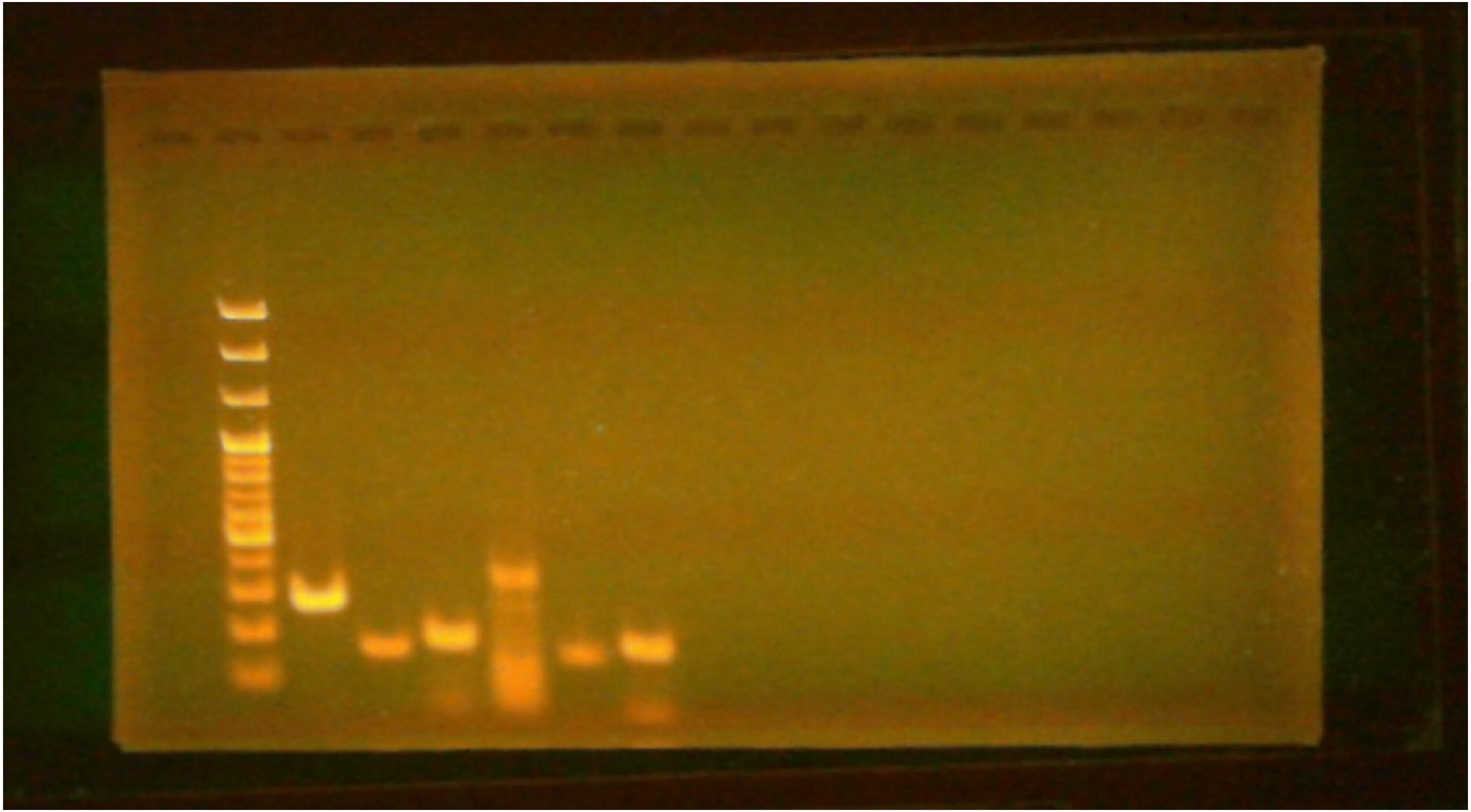

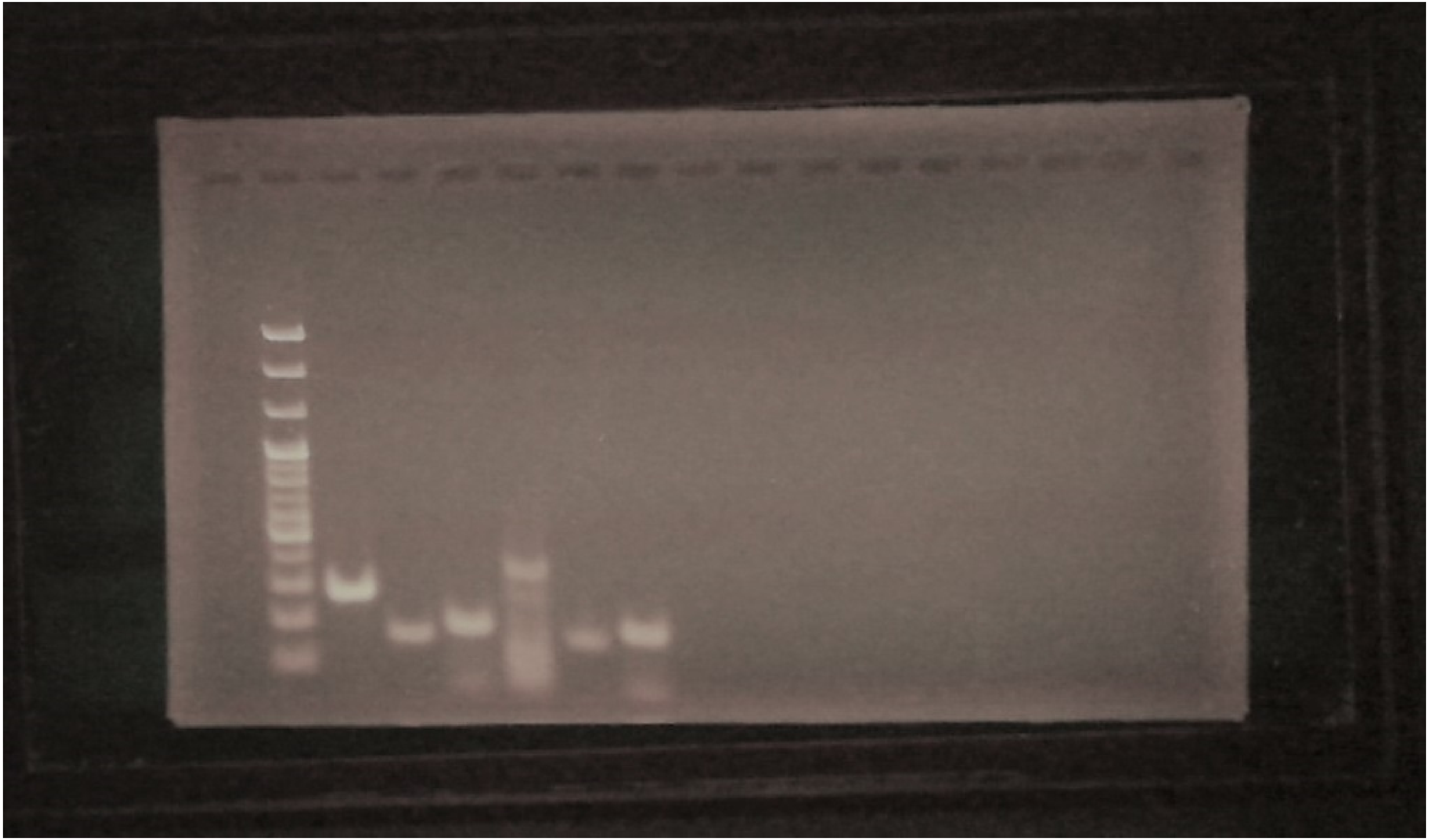

Figure 1. Agarose gel electrophoresis showing PCR amplification of representative *Eimeria* species from field samples. Lane M: 100 bp DNA ladder; Lane 1: *E. tenella* (278 bp) from Mymensingh (Sutiakhali); Lane 2: *E. acervulina* (146 bp) from Mymensingh (Vabokhali); Lane 3: *E. brunetti* (183 bp) from Rajshahi (Puba); Lane 4: *E. mitis* (330 bp) from Comilla (Sarail); Lane 5: *E. maxima* (162 bp) from Dhaka (Savar); Lane 6: *E. necatrix* (162 bp) from Rangpur (Gangachara).
