# Supplementary material for: Molecular detection and risk factors of Eimeria in native and exotic chickens under varying management systems in Bangladesh
Source: PLoS One. 2025 Jul 15;20(7):e0327037. doi: 10.1371/journal.pone.0327037 (PMC12262850; doi:10.1371/journal.pone.0327037)
Supplement: S2_raw_images — (PDF) [file pone.0327037.s002.pdf]

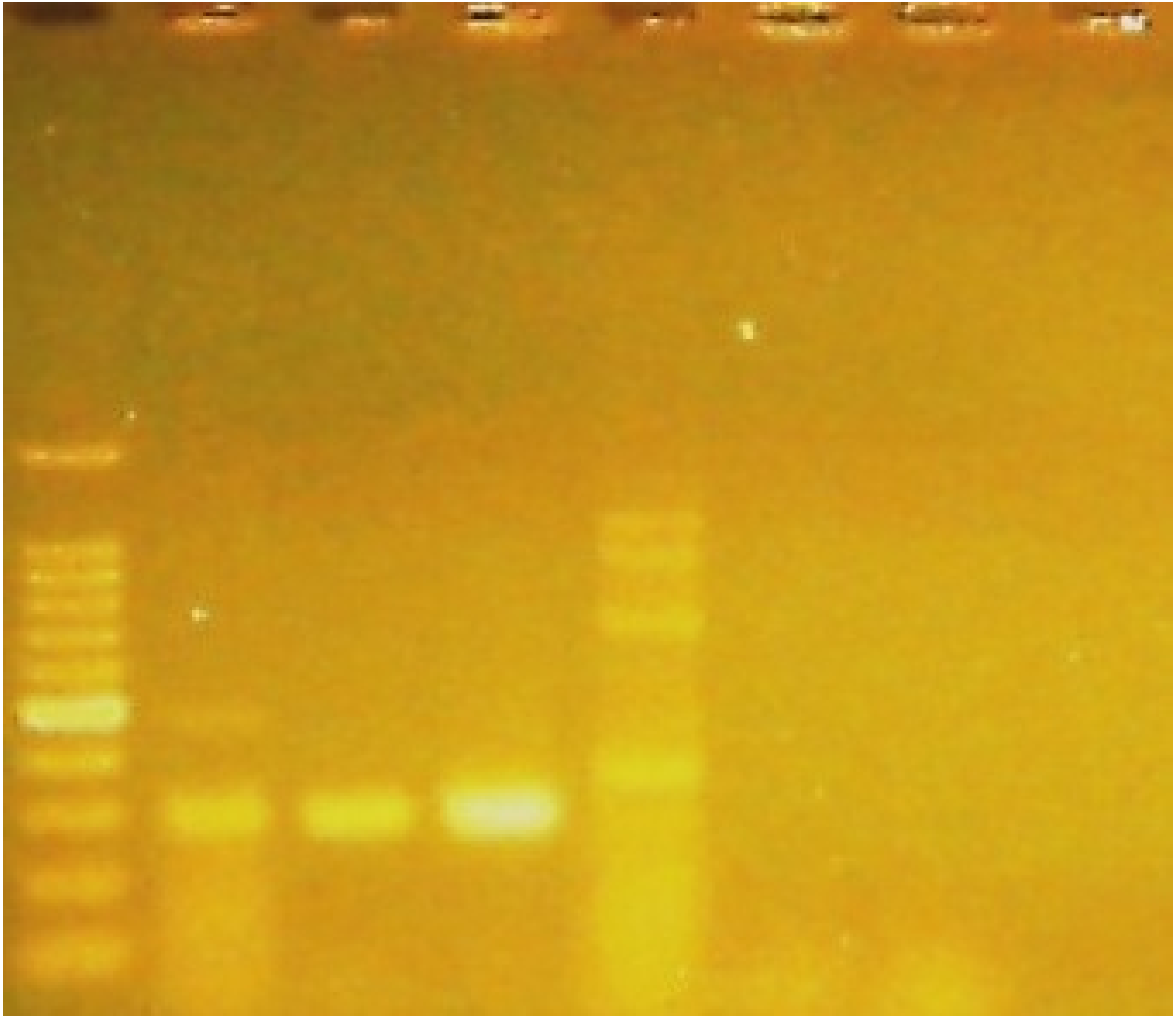

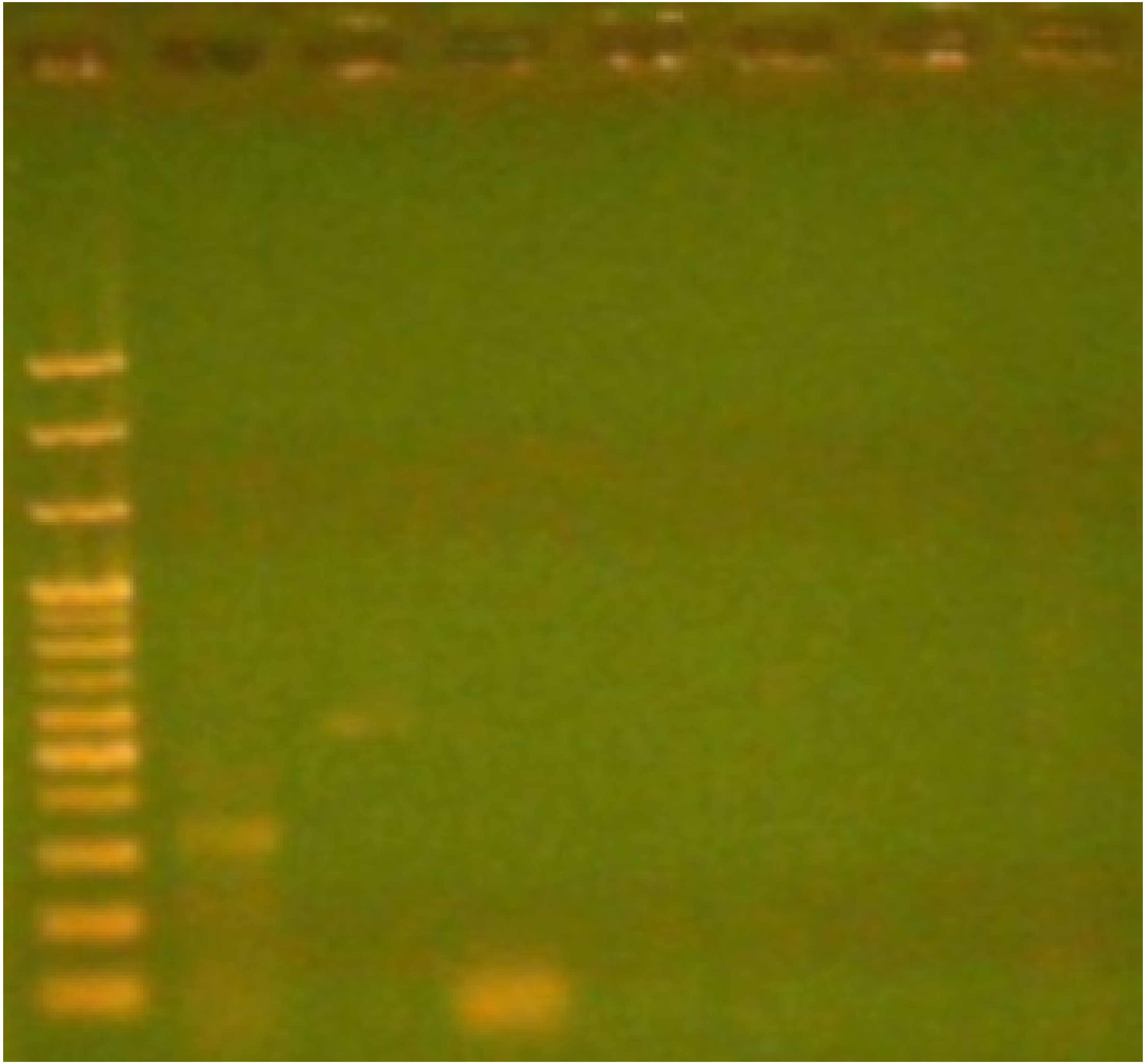

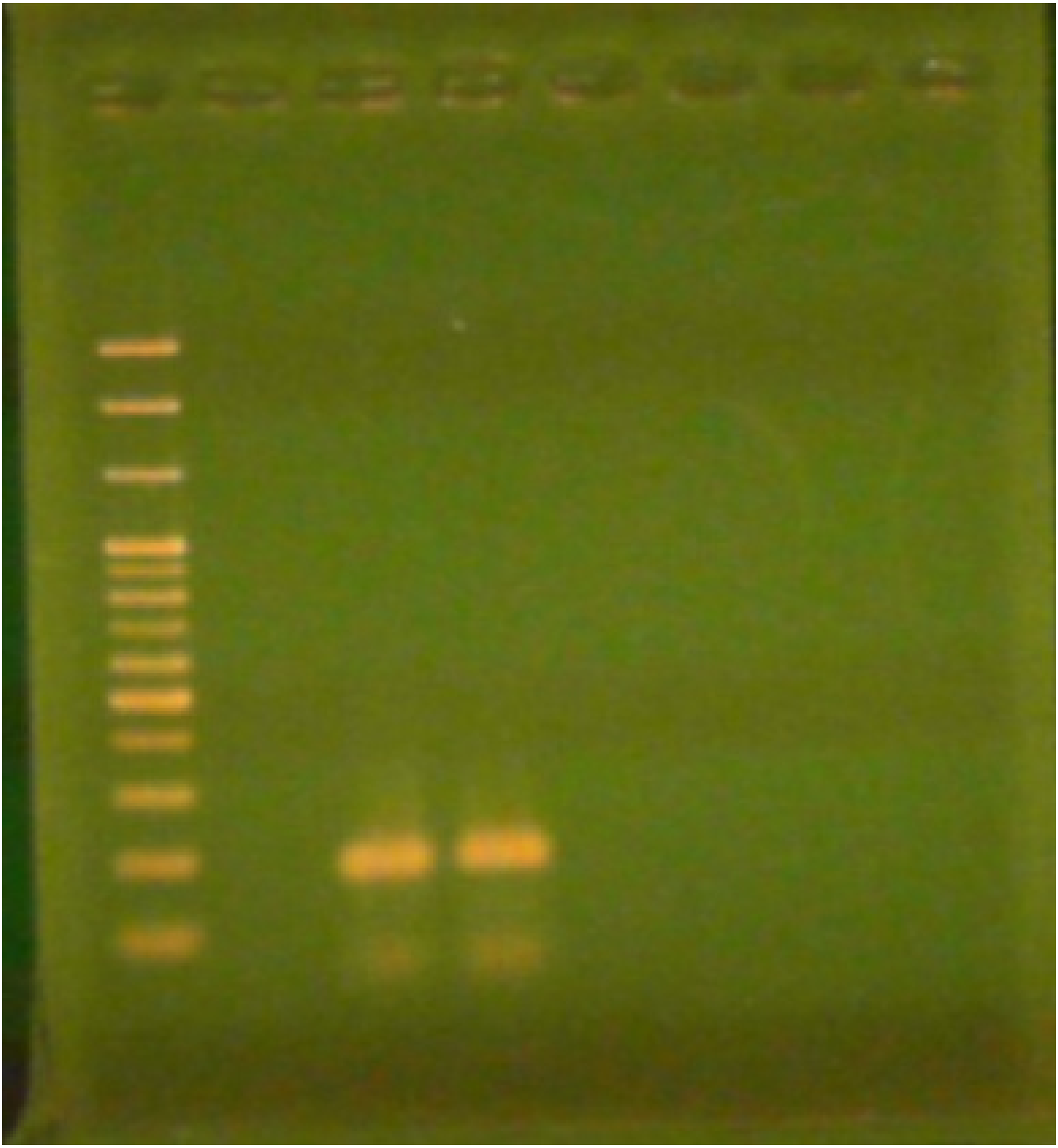

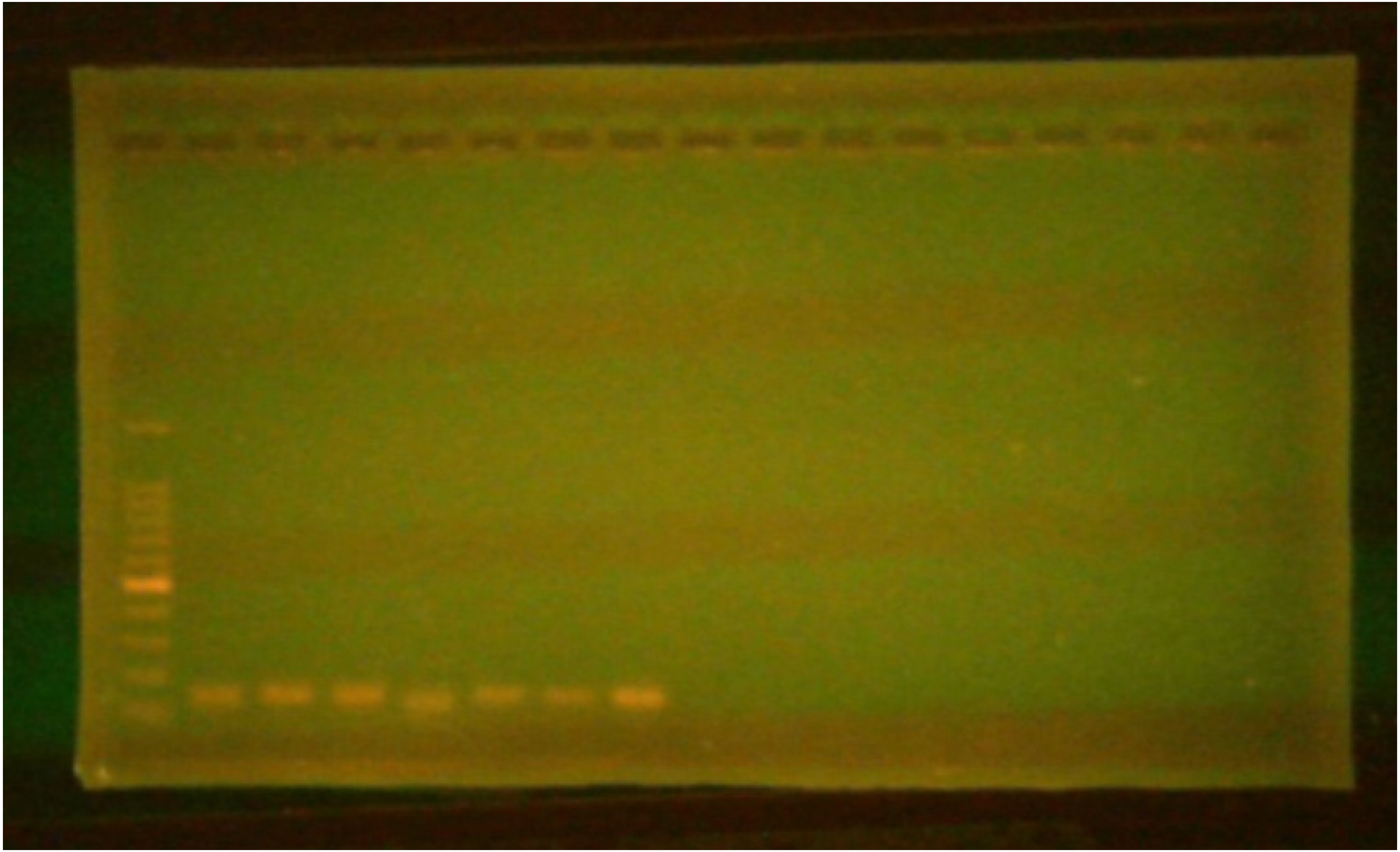

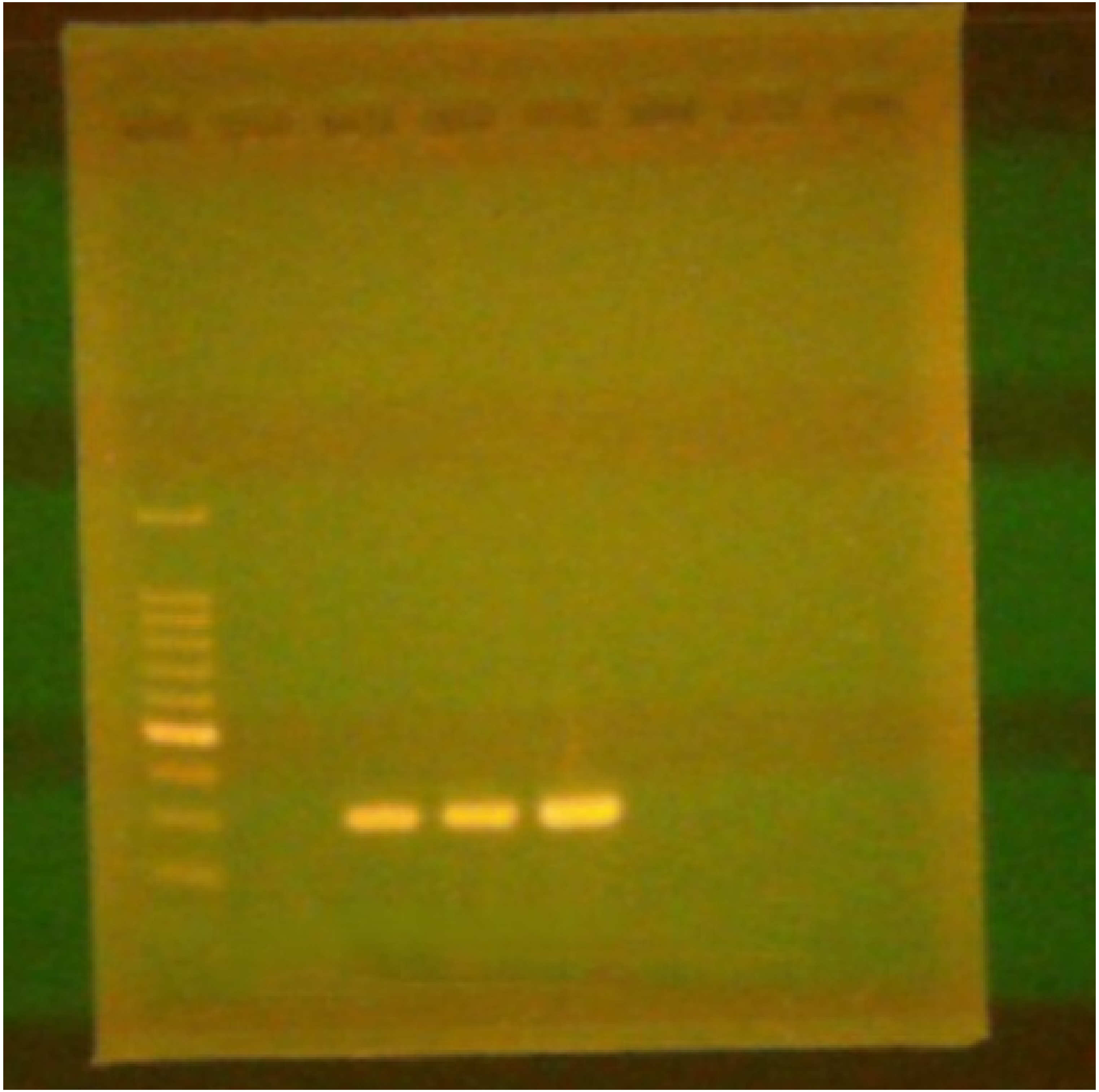

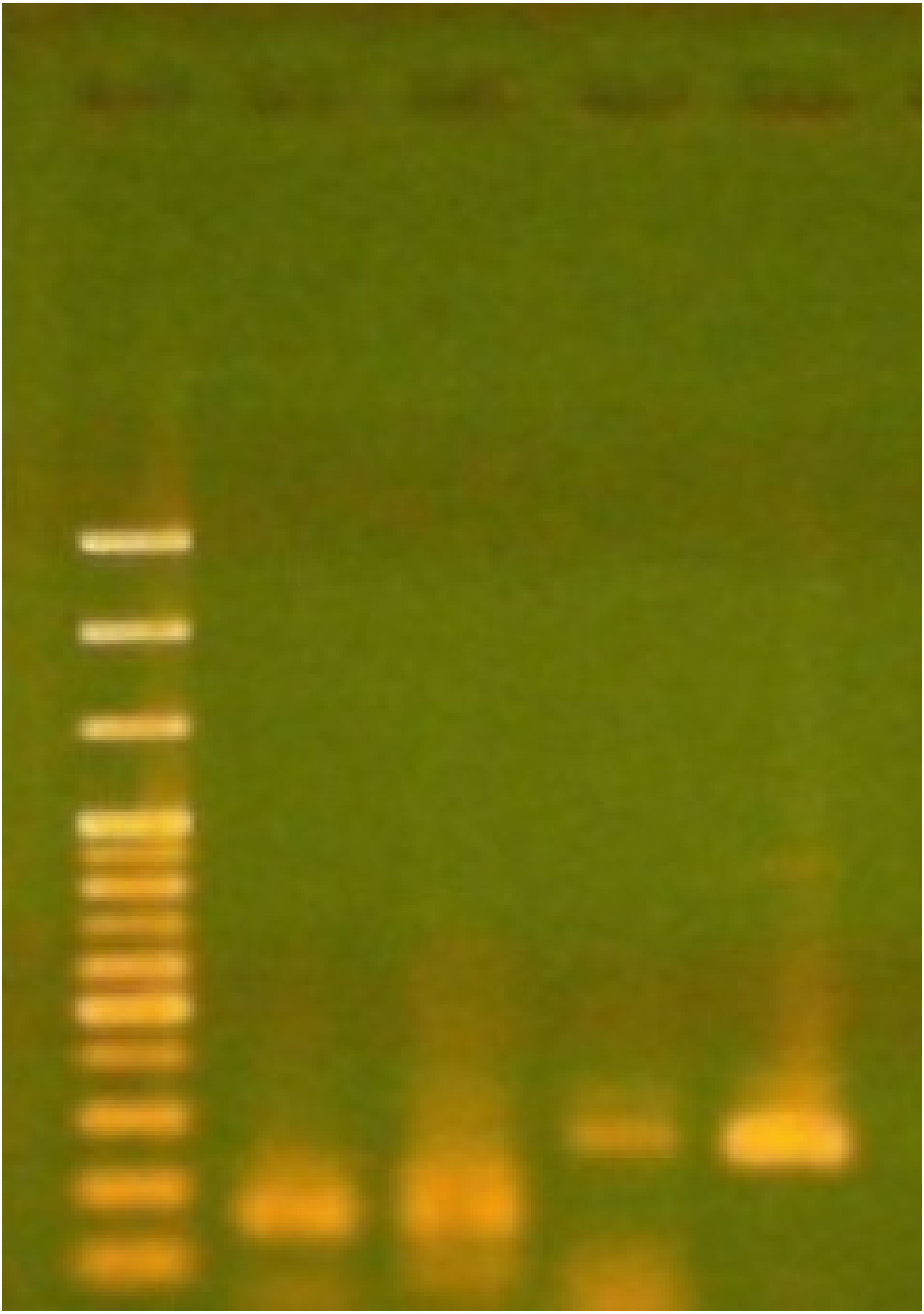

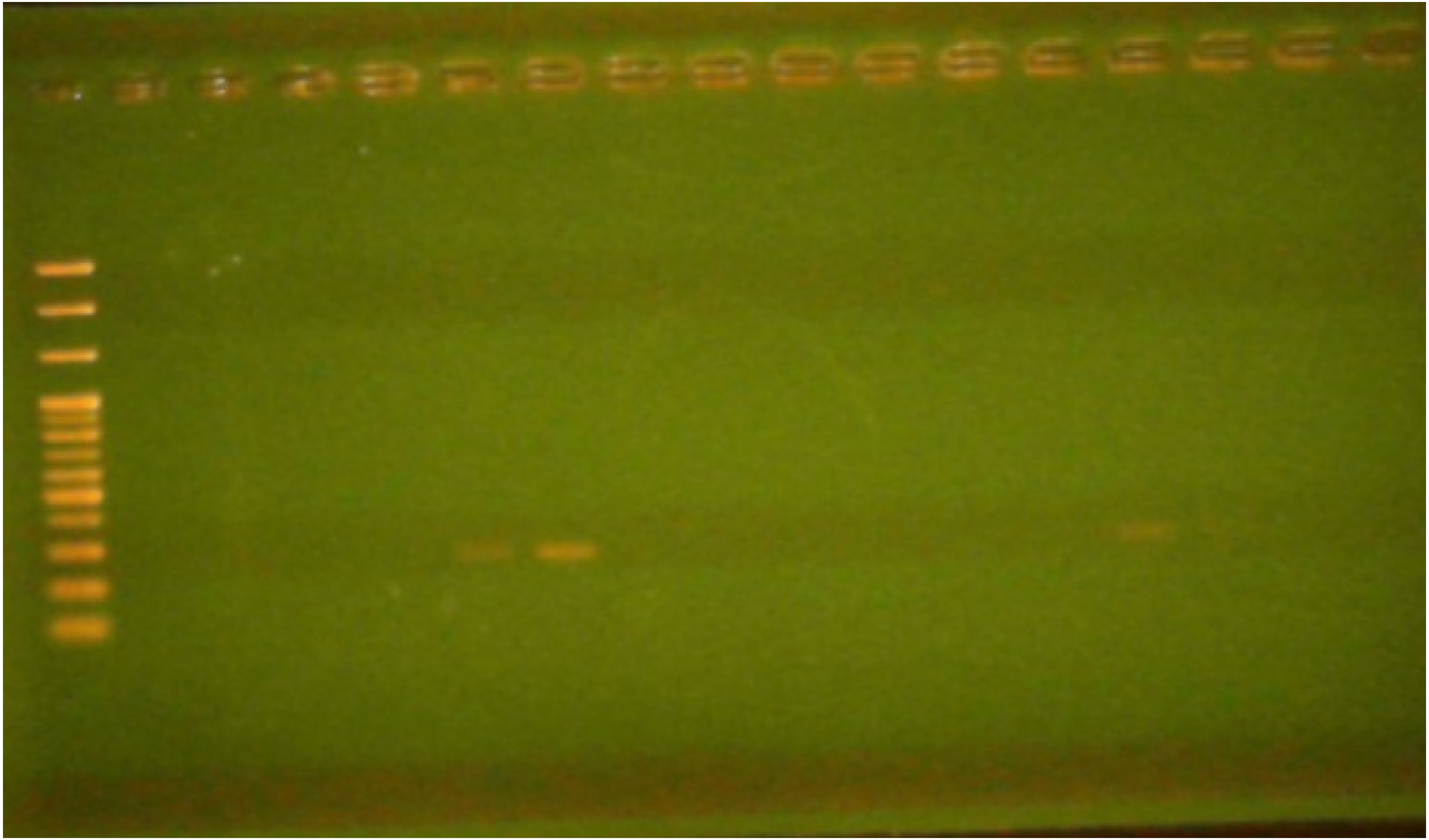

Figure Legend: PCR Detection of *Eimeria* spp. from Field Samples

Page 1: Representative gel image of PCR amplification from fecal samples collected from broiler chickens at Sutiakhali farms, Mymensingh. Lanes 2–4 show positive bands for *Eimeria tenella* (278–300 bp).

Page 2: PCR detection from fecal samples of Sonali chickens collected from Comilla (Sarail). Lane 2 is positive for *Eimeria mitis* (330 bp).

Page 3: PCR results from Sonali chickens in Rajshahi (Puba). Lanes 3–4 are positive for *Eimeria brunetti* (183 bp).

Page 4: Fecal samples from Sonali and broiler chickens collected from two farms in Mymensingh (Vabokhali). Lanes 2–8 show positive bands for *Eimeria acervulina* (146 bp) from Sonali chickens.

Page 5: PCR products from Sonali and broiler chickens in Dhaka (Savar). Lanes 3–5 are positive for *Eimeria maxima* (162 bp).

Page 6: PCR detection from broiler chickens in Rangpur (Gangachara). Lanes 2–3 are positive for *Eimeria necatrix* (162 bp), and lanes 4–5 for *Eimeria tenella* (278–300 bp).

Page 7: Detection of *Eimeria* from Deshi native chickens in Mymensingh (Vabokhali). Lanes 5, 6, and 13 are positive for *Eimeria tenella* (278–300 bp).

Positive bands were excised, gel-purified, and sequenced. Species identity was confirmed through sequence analysis, and representative sequences were submitted to GenBank (accession numbers provided in the manuscript).
