# Supplementary material for: Molecular detection and risk factors of Eimeria in native and exotic chickens under varying management systems in Bangladesh
Source: PLoS One. 2025 Jul 15;20(7):e0327037. doi: 10.1371/journal.pone.0327037 (PMC12262850; doi:10.1371/journal.pone.0327037)
Supplement: Attachment — Submitted filename: Response to Reviewers.docx [file pone.0327037.s004.docx]

**Journal Requirements (Academic Editor):**

When submitting your revision, we need you to address these additional requirements.

1. Please ensure that your manuscript meets PLOS ONE's style requirements, including those for file naming. The PLOS ONE style templates can be found at

<https://journals.plos.org/plosone/s/file?id=wjVg/PLOSOne_formatting_sample_main_body.pdf> and

[https://journals.plos.org/plosone/s/file?id=ba62/PLOSOne_formatting_sample_title_authors_affiliations.pdf](https://track.editorialmanager.com/CL0/https:%2F%2Fjournals.plos.org%2Fplosone%2Fs%2Ffile%3Fid=ba62%2FPLOSOne_formatting_sample_title_authors_affiliations.pdf/1/010f0196d7834d06-77197ca0-be4b-477d-8642-b77c6255dc7b-000000/s_19k2Rt93FPNYly1wKvd2fWiT6cqX7R0z121FwtYeI=211)

**Author Response:**

Thank you for the reminder. We have thoroughly reviewed and revised our manuscript to ensure full compliance with PLOS ONE's style requirements, including formatting, figure legend presentation, and file naming conventions.

2. In your Methods section, please provide additional information regarding the permits you obtained for the work. Please ensure you have included the full name of the authority that approved the field site access and, if no permits were required, a brief statement explaining why.

**Author Response:**

Ethical considerations and permits (The information were added in Methodology section of revised manuscript) Lines 219-238.

Fecal samples were collected non-invasively from chicken farm litter or from the ground surface in homestead free-ranging systems, without any handling or disturbance to the chickens. No animals were restrained, touched, or harmed during the sample collection process. Therefore, ethical approval was not required for this study. All samples were collected with prior verbal consent from farm owners or household heads. Since the study involved environmental sampling from private properties and did not include animal experimentation, no specific government permits were necessary. Field access was granted by the respective owners, and no protected areas or government-owned facilities were involved.

3. We note that the grant information you provided in the ‘Funding Information’ and ‘Financial Disclosure’ sections do not match.

When you resubmit, please ensure that you provide the correct grant numbers for the awards you received for your study in the ‘Funding Information’ section.

**Author Response:**

Thank you for your comment regarding the grant information. I did not find a separate "Financial Disclosure" section in the submission system. However, I located the "Funding Information" section, where I have correctly provided the grant number for the award: BAS-USDA Program/2023/26(12). Please let me know if any further clarification or revision is needed.

4. Thank you for stating the following financial disclosure:
“BAS-USDA Program/2023/26(12)”

Please state what role the funders took in the study. If the funders had no role, please state: "The funders had no role in study design, data collection and analysis, decision to publish, or preparation of the manuscript."
If this statement is not correct you must amend it as needed.
Please include this amended Role of Funder statement in your cover letter; we will change the online submission form on your behalf.

**Author Response:**

The funders had no role in study design, data collection and analysis, decision to publish, or preparation of the manuscript

5. Thank you for stating the following in the Acknowledgments Section of your manuscript:
“This research was funded by the Bangladesh Academy of Science (BAS)-USDA Endowment Program (BAS-USDA Program/2023/26(12)). The authors extend their gratitude to Prof. Dr. Tofazzal Islam, BAS Fellow, for his valuable monitoring of the project.”

We note that you have provided funding information that is not currently declared in your Funding Statement. However, funding information should not appear in the Acknowledgments section or other areas of your manuscript. We will only publish funding information present in the Funding Statement section of the online submission form.
Please remove any funding-related text from the manuscript and let us know how you would like to update your Funding Statement. Currently, your Funding Statement reads as follows:
“BAS-USDA Program/2023/26(12)”

Please include your amended statements within your cover letter; we will change the online submission form on your behalf.

**Author Response:**

We removed the funding-related text from the manuscript (Acknowledgments section). The funding statement will read as “BAS-USDA Program/2023/26(12)”.

6. We note that Figure 1 in your submission contain [map/satellite] images which may be copyrighted. All PLOS content is published under the Creative Commons Attribution License (CC BY 4.0), which means that the manuscript, images, and Supporting Information files will be freely available online, and any third party is permitted to access, download, copy, distribute, and use these materials in any way, even commercially, with proper attribution. For these reasons, we cannot publish previously copyrighted maps or satellite images created using proprietary data, such as Google software (Google Maps, Street View, and Earth). For more information, see our copyright guidelines: <http://journals.plos.org/plosone/s/licenses-and-copyright>.

 We require you to either (1) present written permission from the copyright holder to publish these figures specifically under the CC BY 4.0 license, or (2) remove the figures from your submission:

**Author Response:**

We have removed the figure from submission and deleted the Figure caption and citation in the texts.

7. PLOS ONE now requires that authors provide the original uncropped and unadjusted images underlying all blot or gel results reported in a submission’s figures or Supporting Information files. This policy and the journal’s other requirements for blot/gel reporting and figure preparation are described in detail at <https://journals.plos.org/plosone/s/figures#loc-blot-and-gel-reporting-requirements> and <https://journals.plos.org/plosone/s/figures#loc-preparing-figures-from-image-files>. When you submit your revised manuscript, please ensure that your figures adhere fully to these guidelines and provide the original underlying images for all blot or gel data reported in your submission. See the following link for instructions on providing the original image data: <https://journals.plos.org/plosone/s/figures#loc-original-images-for-blots-and-gels>.

In your cover letter, please note whether your blot/gel image data are in Supporting Information or posted at a public data repository, provide the repository URL if relevant, and provide specific details as to which raw blot/gel images, if any, are not available. Email us at [plosone@plos.org](mailto:plosone@plos.org) if you have any questions.

**Author Response:**

The original uncropped and unadjusted images are uploaded as a Supporting Information file 'S1_raw_images' (PDF) and 'S2_raw_images'

**Response to Reviewers**

Reviewer #1: The study ‘Molecular Detection and Risk Factors of Eimeria in Native and Exotic Chickens under Varying Management Systems in Bangladesh’ was conducted by Dr. Rahman and its groups, and reported a variety of interesting results and providing valuable information to relevant poultry coccidiosis fields.

I have read the paper wholeheartedly, and here are some questions:

Reviewer Comment:

L58-60/Figure 1: You mentioned in the introduction that Bangladesh's hot and humid climate provides favorable conditions for the development of Eimeria, but your measured risk factors do not provide any temperature, humidity, or rainfall data.
Why didn't you measure the key elements of Eimeria development mentioned in the introduction and use them as risk factors?

**Author Response:**

Thank you for this thoughtful observation. We fully acknowledge that climatic variables such as temperature, humidity, and rainfall are critical to the sporulation and survival of *Eimeria* oocysts and are thus key factors influencing the epidemiology of coccidiosis. Our mention of Bangladesh’s hot and humid climate in the introduction was intended to provide contextual background, as these general environmental conditions are known to create a conducive setting for the parasite’s development.

However, the current study was designed as a cross-sectional field survey focusing on breed susceptibility, management practices, feeding systems, and rearing conditions as primary risk factors. Given the resource and logistical constraints, real-time and location-specific meteorological data were not collected during sampling. Moreover, since sampling occurred over diverse regions and time frames, integrating consistent and reliable climatic data across all sites would have required a separate longitudinal study design and collaboration with national meteorological services.

Instead, we used proxy indicators, such as litter management, housing type, and production system (intensive vs. free-range), which are closely linked to micro-environmental conditions within poultry facilities and have direct relevance to *Eimeria* transmission dynamics.

We appreciate your suggestion and agree that integrating climatic variables would enhance our understanding of *Eimeria* epidemiology. We plan to consider this in future longitudinal studies with geospatial mapping and climatic overlays to better capture environmental influences on infection risk.

Reviewer Comment:

L415-417: The question I want to ask the most is what the meaning of this comprehensive study is, which is already written in the limitations of the study.
Even if various positive samples were found in different regions of Bangladesh, unless data on the phenotype represented by the chickens, e.g., lesion score, are submitted, this study feels inconclusive.
In addition, as you have already mentioned, no relevant data have been submitted on whether oocysts actually exhibit clinically pronounced infection symptoms, even if they are found.
Therefore, I think this study you conducted is insufficient to draw any conclusions despite the time and effort it took.

**Author Response:**

We sincerely thank the reviewer for this critical and insightful comment. We agree that clinical phenotyping, such as lesion scoring and the documentation of symptomatic infections, would significantly strengthen the implications of Eimeria detection and contribute to understanding its true pathological impact.

However, the primary objective of this study was to provide the first comprehensive baseline data on the prevalence and species diversity of *Eimeria* spp. across a wide geographical range and among different chicken breeds and management systems in Bangladesh using both parasitological and molecular methods. Prior to this study, data on *Eimeria* infection status especially among native chickens were extremely limited or nonexistent in many regions. By detecting oocysts and identifying six *Eimeria* species (including *E. tenella* and *E. acervulina* as dominant), our findings help define the distribution and breed susceptibility patterns under existing field conditions.

We acknowledge that the absence of lesion scoring, clinical symptom tracking, and histopathological assessments limits conclusions about clinical severity or economic impact, and we clearly stated this in the Limitations section. Still, the identification of oocyst-positive samples across regions and rearing systems is an essential first step to inform future targeted studies that can integrate phenotypic and pathological assessments.

This foundational survey also provides crucial epidemiological context for native vs. exotic breed vulnerability, risk factor associations (e.g., feed type and production system), and the molecular diversity of *Eimeria* in Bangladesh. These results are already being used to guide ongoing longitudinal and intervention-based research, including work incorporating clinical assessments and anticoccidial resistance monitoring.

While we agree that further work is needed to build on these findings, we believe this study offers meaningful initial insights into *Eimeria* epidemiology in Bangladesh and establishes a baseline for future, more clinically detailed investigations.

Reviewer Comment:

L639: In this table format, I cannot understand which one has more risk factors than the others.
It can be seen that there was a significant difference between the two categories.
It would be good to choose a different statistical method or use a method that can be indicated.

**Author Response:**

We thank the reviewer for this valuable comment. We agree that while Table 2 presents detailed descriptive data on prevalence across multiple risk factor categories (e.g., breed, age, sex, feed type, and rearing system), it lacks a statistical summary that clearly communicates the relative strength and significance of these risk factors.

In response to this suggestion, we have taken the following actions to improve clarity and interpretation:

We have now conducted a chi-square test (χ²) for categorical variables (e.g., breed group, rearing system, feed type, sex, and age category) to determine the statistical significance of associations between these risk factors and *Eimeria* prevalence. Significant differences (p < 0.05) are now clearly indicated in the revised table and in the main text (Lines 262-264; 301-302).

To improve readability, we have redesigned Table 2 to clearly distinguish between statistically significant and non-significant categories. Highlight high-risk categories in bold or with asterisks (*p < 0.05).

We appreciate the reviewer’s feedback, which helped us improve the analytical robustness and clarity of the manuscript.

Reviewer Comment:

L648: A positive sample itself cannot mean serious clinical symptoms.
Beyond just a sample showing positive or negative, is there an experimentally infected control group?
For example, have you confirmed that similar band sizes and locations occur in chickens that have been orally administered Eimeria enough to cause clinical symptoms?

**Author Response:**

We sincerely thank the reviewer for this insightful comment. We fully agree that a positive PCR result or the presence of *Eimeria* oocysts in fecal samples does not necessarily indicate clinically significant coccidiosis, particularly in the absence of lesion scoring, clinical signs, or production losses.

As stated in the limitations of our study, this was a cross-sectional, field-based investigation focusing on the natural occurrence and molecular identification of *Eimeria* spp. under different management systems in Bangladesh. Because of the non-invasive nature of our sampling approach—collection of naturally shed feces from the environment—we did not include an experimentally infected control group or conduct clinical evaluations such as lesion scoring or growth performance monitoring.

We acknowledge that experimental infection studies, where birds are orally challenged with known doses of *Eimeria* spp. and monitored for clinical symptoms and lesion development, are essential to establish a direct link between molecular findings and pathogenicity. However, our study was designed to characterize field prevalence and species distribution rather than assess disease severity under controlled conditions.

To ensure species-specific identification, we used validated primers, and positive amplicons were gel-purified and sequenced. The identity of the *Eimeria* species was confirmed via sequence analysis, with representative sequences submitted to GenBank (accession numbers provided in the manuscript).

We appreciate this valuable suggestion and plan to address this important aspect in future studies through controlled infection experiments and longitudinal field trials that incorporate clinical assessments and lesion scoring to correlate molecular findings with pathogenic outcomes.

**Comments:**

Table 2: Typo: Brioler > broiler

**Response:** Done

Table 3: Typo in primer sequence 'CWCACCACTCACAATGAGGCAC'

**Response:** Thank you for pointing this out. We have carefully rechecked the primer sequence ‘CWCACCACTCACAATGAGGCAC’ and confirm that it is correct, as originally described by Haug et al. (2007). These primers are specific for the short variant of the ITS-1 region (*GenBank* accession no. AF065095), as cited in our manuscript.

Table 5: Typo: Brioler > broiler

**Response:** Done

Reviewer #2: This cross-sectional study provides valuable epidemiological data on Eimeria spp. prevalence in Bangladeshi chickens, combining field surveys with molecular characterization. The large sample size (n=1,500) and multi-district approach strengthen the findings. The identification of commercial feed and intensive systems as risk factors offers practical implications for poultry management. However, methodological details require clarification to ensure reproducibility, and some analytical aspects need expansion. The work would benefit from improved technical descriptions and better integration between methodology and results.

1. Sample Collection Methodology:
The fecal sample collection protocol lacks critical details. Please specify: Number of samples collected per farm/flock.
Minimum fecal quantity per sample (grams). Clarify whether pooled samples were used or individual bird samples were maintained separately.

**Response:** Thank you for suggesting the critical details for methodology section. We have provided the no of samples (15-25) collected per farm/flock. The amount of fecal quantity (7-10g) per sample. We have used individual bird samples (Line no. 136-138 and 140-141)

2. Oocyst Detection Protocol (Major):
The salt flotation method requires validation details. State whether McMaster chamber counts followed standard OPG calculations (oocysts per gram). Cite original references for both flotation and McMaster techniques

**Response:**

Thank you for your valuable comment. We have revised the "Oocyst Detection Protocol" section to include validation details of the salt flotation method and to clarify that McMaster chamber counts were used to calculate standard oocysts per gram (OPG) values (Line no. 153 to 158). The original references for both the flotation method and the McMaster counting technique have been appropriately cited (Reference no. 18).

3. Molecular Methods (Major):
PCR protocols need technical enhancement. Specify synthesis source (company) and purification grade. State primer concentrations in master mixes. Include positive/negative controls used. Clarify species differentiation method: Were species-specific primers used, or was sequencing performed?

**Response:**
We thank the reviewer for these constructive suggestions to improve the clarity and technical accuracy of our molecular methods section. In response, we have revised the relevant section in the manuscript to include the following enhancements:

- **Primer synthesis source and purification grade:** All primers used in this study were synthesized by Macrogen Inc., Korea, and were MOPC-purified to ensure high specificity and quality for PCR amplification.
- **Primer concentrations in PCR master mix:** PCR reactions were performed in a final volume of 25 µL, containing 12.5 µL of 2× PCR Master Mix (Takara, Japan), 0.5 µM of each forward and reverse primer, 2 µL of template DNA, and nuclease-free water to adjust the final volume.
- **Controls:** Positive controls included DNA extracted from field isolated *Eimeria* species confirmed by sequence analysis. Negative controls consisted of PCR reactions with nuclease-free water in place of template DNA. These controls were included during PCR run to validate the amplification results and rule out contamination.
- **Species differentiation method:** Species-specific primers targeting the internal transcribed spacer-1 (ITS-1) region were used for initial PCR detection of *Eimeria* spp., as described by Haug et al. (2007). To confirm the identity of the PCR products, representative amplicons were excised from the gel, purified, and subjected to Sanger sequencing using forward and reverse primers. The obtained sequences were aligned with known sequences in GenBank using BLAST to confirm species identity. Accession numbers of the confirmed sequences have been provided in the manuscript (LC855064–LC855069).

We have updated the **Materials and Methods** section of the manuscript accordingly to reflect these details and improve reproducibility (Lines no 172-179 and 200-201).

4. Figure 2 Concerns (Major): The gel electrophoresis image requires, Clear lane labels indicating sample types (test samples vs controls). Provide representative gel images of clinical samples (not just positive controls) in the main text or supplementary materials.

**Response:**

Thank you for your valuable feedback. We acknowledge the need for clear labeling indicating samples types. In response to your comment, we have made the following revisions:

1. **Lane Labeling**: We have updated Figure 2 caption (Now Figure 1) to include clear lane labels, indicating the test samples (field isolates). (Lines 187 – 192)
2. **Representative Clinical Samples**: The representative gel image of PCR amplification products from actual field samples collected from different geographical locations, not just positive controls have been provided in the supplementary materials (S1_raw_images and S2_raw_images).

We believe these revisions enhance the clarity and scientific rigor of our findings and sincerely appreciate your helpful suggestions.

5. Resolve discrepancy between methods mentioning phylogenetic analysis and missing results, Either present the evolutionary tree with bootstrap values.

**Response:**

Thank you for your insightful observation. We acknowledge the discrepancy between the Methods section, which initially mentioned phylogenetic analysis, and the absence of corresponding results in the manuscript.

Upon further consideration, we decided to remove the phylogenetic analysis due to limited sequence variation among the amplified regions, which did not yield meaningful evolutionary differentiation among the Eimeria species in our dataset. Therefore, the reference to phylogenetic analysis has been deleted from the Methods section to maintain consistency and avoid confusion.

We appreciate your careful review and understanding.
